# Altered Cerebro-Cerebellar Limbic Network in AD Spectrum: A Resting-State fMRI Study

**DOI:** 10.3389/fncir.2019.00072

**Published:** 2019-11-06

**Authors:** Zhigang Qi, Yanhong An, Mo Zhang, Hui-Jie Li, Jie Lu

**Affiliations:** ^1^Department of Radiology, Xuanwu Hospital, Capital Medical University, Beijing, China; ^2^Beijing Key Laboratory of Magnetic Resonance Imaging and Brain Informatics, Beijing, China; ^3^Key Laboratory of Behavioral Science, Institute of Psychology, Chinese Academy of Sciences, Beijing, China

**Keywords:** mild cognitive impairment, Alzheimer’s disease, cerebro-cerebellar connectivity, limbic network, resting-state functional MRI, compensation

## Abstract

Recent evidence suggests that the cerebellum is related to motor and non-motor cognitive functions, and that several coupled cerebro-cerebellar networks exist, including links with the limbic network. Since several limbic structures are affected by Alzheimer pathology, even in the preclinical stages of Alzheimer’s disease (AD), we aimed to investigate the cerebral limbic network activity from the perspective of the cerebellum. Twenty patients with mild cognitive impairment (MCI), 18 patients with AD, and 26 healthy controls (HC) were recruited to acquire Resting-state functional MRI (rs-fMRI). We used seed-based approach to construct the cerebro-cerebellar limbic network. Two-sample *t*-tests were carried out to explore the differences of the cerebellar limbic network connectivity. The first result, a sub-scale network including the bilateral posterior part of the orbitofrontal cortex (POFC) extending to the anterior insular cortex (AIC) and left inferior parietal lobule (L-IPL), showed greater functional connectivity in MCI than in HC and less functional connectivity in AD than in MCI. The location of this sub-scale network was in accordance with components of the ventral attention network. Second, there was decreased functional connectivity to the right mid-cingulate cortex (MCC) in the AD and MCI patient groups relative to the HC group. As the cerebellum is not compromised by Alzheimer pathology in the prodromal stage of AD, this pattern indicates that the sub-scale ventral attention network may play a pivotal role in functional compensation through the coupled cerebro-cerebellar limbic network in MCI, and the cerebellum may be a key node in the modulation of social cognition.

## Introduction

The most prominent feature of Alzheimer’s disease (AD) is the compromise of episodic memory, even in its prodromal stage that is referred to as mild cognitive impairment (MCI). It is natural that the pathogenesis of the impairment of memory became a focus on the research of AD. Although it is also involved in memory function, the limbic system has attracted little attention in AD-related research (Rolls, 2015). However, the common neuropsychiatric symptoms (NPS) of AD, including agitation and aggression, can result in potential harm that affects the patients and their caregivers and may become a much more serious burden on the family than the amnesia (Craig et al., 2005). For most psychiatric conditions, dysfunction of the limbic structures affects emotion regulation, social interaction, and other behaviors. Understanding the limbic network would be meaningful for deeply probing brain functions, such as memory, and for clarifying the pathogenesis of psychiatric disorders, such as depression (Bennett, 2011), bipolar disorder (Leow et al., 2013), and psychosocial stress (Pruessner et al., 2008). To date, very few reports have focused on the significance of the limbic network in the evaluation of the biological underpinnings of AD (Trzepacz et al., 2013).

A series of clinical studies exploring NPS in patients with MCI and AD indicated that greater agitation was correlated with decreased gray matter (GM) density in the left insula and bilateral anterior cingulate (Bruen et al., 2008) and the left inferior frontal, insular, and bilateral retrosplenial cortices (Hu et al., 2015). Similarly, Trzepacz et al. (2013) reported that agitation and aggression severity was positively correlated to frontolimbic atrophy. Limbic dysfunction mainly includes the entorhinal cortex extending to the parietal cortex (Khan et al., 2014) and inferior frontal areas. Several limbic structures are involved by tau pathology even in AD patients (Spires-Jones and Hyman, 2014). With combined positron emission tomography (PET) and magnetic resonance imaging (MRI), severe reductions of metabolism were observed throughout a network of limbic structures, including the hippocampus, medial thalamus, and posterior cingulate cortex (PCC) in mild AD patients; the same pattern was seen in amnestic MCI to a lesser degree (Nestor et al., 2003).

Resting-state functional MRI (rs-fMRI) can be used to evaluate the functional connectivity and large-scale functional network such as the default mode network (DMN) and limbic network. A typical finding is impairment of DMN activity, which is considered to be the origin of episodic memory damage in MCI and AD (Greicius et al., 2004; Li et al., 2015). Rs-fMRI may be particularly useful in the early detection of pathological change, due to neural function alterations may precede neuronal atrophy. Since the structure of the limbic system is compromised in MCI and AD, it is reasonable to hypothesize that functional changes of the limbic system occur in AD, even in the prodromal stages. A resting-state magnetoencephalography study on patients with MCI due to AD showed that patients with phosphorylated tau pathology had decreased functional connectivity such as the PCC, orbitofrontal cortex (OFC), and paracentral lobule, which could affect the limbic structures (Canuet et al., 2015).

The cerebellum has long been considered as mainly being involved in motor function; recently, it was also found to contribute to some non-motor cognitive functions. In other words, in addition to sensorimotor function, cognition, emotion, and autonomic functions can also be localized to the cerebellum, just like with the cerebrum. The cognitive/limbic cerebellum is found to be located in the cerebellar posterior lobe, which has been connected to cerebral cortex association areas. Moreover, lesions in the cerebellar posterior lobe lead to the cerebellar cognitive affective syndrome (CCAS). New evidence regarding cerebellar organization and functional connections have been provided by rs-fMRI studies in humans that have found distributed cerebral networks that underlie movement, attention, and limbic valence, as well as frontoparietal and default systems concerned with multiple different functions map onto the cerebellum with topographic specificity (Habas et al., 2009; O’Reilly et al., 2010). Patients with focal infarcts to the hemipons, present disrupted functional coupling between the cerebrum and contralateral cerebellum (Lu et al., 2011). Buckner et al. (2011) proposed an rs-fMRI-based approach to comprehensively explore the organization of cerebro-cerebellar circuits in the human brain. Seven networks, including the limbic network, within the cerebellum were observed to connect to the associated cerebral networks.

Because cerebellum plays crucial roles in higher cortical functions through a cerebro-cerebellar circuit, and it is not compromised by AD-related pathology in the early stages (Braak and Braak, 1991), here, we wanted to evaluate the pattern of cerebral limbic network activity of the AD spectrum through the coupled cerebro-cerebellar network.

## Materials and Methods

### Participants Recruitment

Twenty patients with MCI, 18 patients with AD, and 26 healthy controls (HC) were recruited in the study. The MCI and AD participants were recruited from the memory clinic of the Department of Neurology in hospital. HC were enrolled through volunteer posters from a community-based epidemiological study. Participants all provided written informed consent in accordance with the guidelines set by the Medical Research Ethics Committee of Beijing Xuanwu Hospital.

### Clinical Examination

For each subject, clinical examination was composed of medical history, neurological examination, informant interview, and neuropsychological assessment including the Mini-Mental State Examination (MMSE) and Clinical Dementia Rating (CDR). Potential participants with a history of stroke, drug abuse, moderate to serious hypertension, psychiatric diseases, or other systemic diseases were excluded from the study.

HC did not have any subjective or reported cognitive impairments, and a CDR score of 0 and MMSE score ≥28. The inclusion criteria for MCI patients were based on previous studies (Winblad et al., 2004; Zhang et al., 2017) and were as follows: had a subjective cognitive complaint (corroborated by an informant), episodic memory deficit on neuropsychological testing (CDR score = 0.5 and MMSE score >24), and could complete daily living independently. AD patients met both the DSM-IV criteria for dementia and the National Institute of Neurological and Communicative Diseases and Stroke/AD and Related Disorders Association criteria for probable AD dementia.

The demographic and neuropsychological findings of the AD, MCI, and HC groups are summarized in Table 1. Age, gender ratio, and years of education were matched across the three groups. The age of participants was similar between the three diagnostic groups (one-way ANOVA, *F* = 0.590, *p* = 0.559) with similar medians and ranges, whereas the MMSE scores were significantly different between the three groups (one-way ANOVA, *F* = 78.552, *p* < 0.0001).

**Table 1:** Demographics of Alzheimer’s disease (AD) and mild cognitive impairment (MCI) patients, and healthy controls (HC).

	Age	Female/Male	Education (year)	MMSE	CDR
AD patients (*n* = 20)	73.1 ± 6.7	11/9	10.5 ± 0.6	17.5 ± 0.1	1
MCI patients (*n* = 18)	70.5 ± 6.3	10/8	12.3 ± 1	26.1 ± 0.6	0.5
Healthy controls (*n* = 26)	71.3 ± 6.8	12/14	11 ± 0.9	28.3 ± 0.5	0

*No significant difference (p > 0.05) was observed in age, sex, or years of education between the three groups. Significant differences in Mini-Mental State Examination (MMSE) scores were seen between the three groups (p < 0.0001)*.

### MRI Data Acquisition

All MRI data were acquired using a 3-T Siemens Trio system. Participants’ heads were positioned within a 12-channel head coil. Foam padding was provided for comfort and to minimize head movement. During the rs-fMRI scanning, all participants were informed to close their eyes and to restrain from initiating attention-demanding activity. Functional images were collected using a gradient echo sequence [echo time (TE) = 40 ms, repetition time (TR) = 2,000 ms, flip angle = 90°, field of view (FoV) = 256 mm^2^, matrix = 64 × 64, 28 slices, slice thickness = 4 mm, and 0 mm inter-slice gap] for a period of 8 min and 4 s, resulting in a total of 239 imaging volumes. A T1-weighted anatomical image was also obtained using a magnetization-prepared rapid acquisition gradient echo sequence [TE = 2.2 ms, TR = 1,900 ms, inversion time (TI) = 900 ms, flip angle = 9°, FoV = 256 mm^2^, matrix = 224 × 256, 176 slices, and 1 mm^3^ voxel]. 3D T1 structural images were collected for anatomical co-registration.

### MR Image Preprocessing and Individual Limbic Network Mapping

We preprocessed all MRI data using the Connectome Computation System pipeline consisting of anatomical and functional image processing steps (Xu et al., 2015). The anatomical preprocessing firstly denoised individual MRI structural images with a spatially adaptive non-local means filter (Xing et al., 2011). It stripped the skull of the denoised image and integrated manual edits to achieve a better brain extraction and segmented the brain volume into cerebrospinal fluid (CSF), white matter (WM), and GM tissues across both cerebral cortex and cerebellum. Individual pial (GM/CSF boundary) and white (GM/WM boundary) surfaces were subsequently generated and spatially normalized to match a group-level template surface in Montreal Neurological Institute (MNI) space. The subsequent functional preprocessing removed the first five volumes (10 s), detected and fixed temporal spikes by interpolation, corrected temporal acquisition difference in slice order and spatial head motion across volumes and performed intensity normalization on the 4D global mean intensity of 10,000. This pipeline uses individual white surfaces to complete a boundary-based registration (BBR) for building spatial matching between multimodal (functional vs. anatomical) images in a single individual. Friston’s 24-parameter motion curves, WM and CSF mean time series, as well as linear and quadratic trends were regressed out from the individual rs-fMRI time series using multiple linear regressions. As the final step in the CCS, the rs-fMRI time series were transferred into a 1-mm MNI surface grid and down-sampled to the 4-mm MNI surface grid (fsaverage5).

Seed-based method was employed to construct a cerebro-cerebellar limbic network. In this study, seed was chosen following these steps: first, cerebellar limbic network template was chosen from the 7-network parcellation of the cerebellum using 1,500 subjects (Buckner et al., 2011); second, the average time sequence of all the vertex in the template was extracted. Pearson’s correlation coefficient between the individual mean time series of the cerebellar limbic network and preprocessed rs-fMRI time series of each vertex on the fsaverage5 was calculated and further converted into Fisher-*z* value to quantify the limbic functional connectivity. This resulted in individual surface mapping of the limbic network connectivity for subsequent statistical tests across the three groups.

### Correlational Analysis

Two-sample *t*-tests were applied to explore cerebellar limbic network connectivity differences between each pair of the three groups. Vertex-wise statistical cortical surface maps were corrected for multiple comparisons with cluster-level random field theory of family-wise errors (corrected *p* < 0.05). Correlations between MMSE score and mean cerebellar limbic network connectivity across all vertices within each cluster exhibiting significant differences in functional connectivity were performed.

## Results

### Cerebral Areas Showing Significant Functional Connectivity to Cerebellar Limbic Network

**Figure 1:**
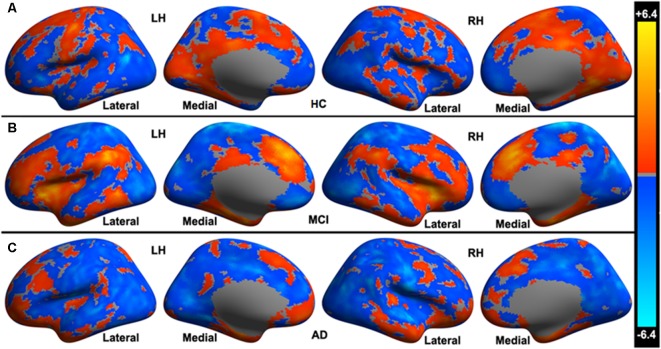
Cerebral areas showing significant functional connectivity to the cerebellar limbic network in **(A)** healthy controls (HC), **(B)** mild cognitive impairment (MCI), and **(C)** Alzheimer’s disease (AD) groups.

In HC, a set of distributed areas showed positive functional connectivity to the cerebellar limbic network, including the frontal lobe, parietal lobe, and temporal lobe, mainly in the medial part of the hemispheres. Some areas also showed negative functional connectivity to the cerebellar limbic network, distributing in the convexity of the hemispheres (see Figure 1A). In the MCI group, fewer cerebral areas showed positive functional connectivity to the cerebellar limbic network than in HC; however, the *t*-values were higher in some areas on visual inspection, mainly in the frontoparietal lobes (see Figure 1B). Many fewer areas exhibited positive functional connectivity to the cerebellar limbic network in AD than in MCI and HC; these areas were mostly distributed in the frontal and temporal pole (see Figure 1C). At the same time, the number of areas showing negative functional connectivity to the cerebellar limbic network was higher in MCI and AD groups than in the HC. These areas showing functional connectivity to the cerebellar limbic network were nearly symmetrically distributed in bilateral hemispheres.

### Significant Differences of the Functional Connectivity Between Cerebellar Limbic Network and Cerebral Cortex Between HC, MCI, and AD Groups

**Figure 2:**
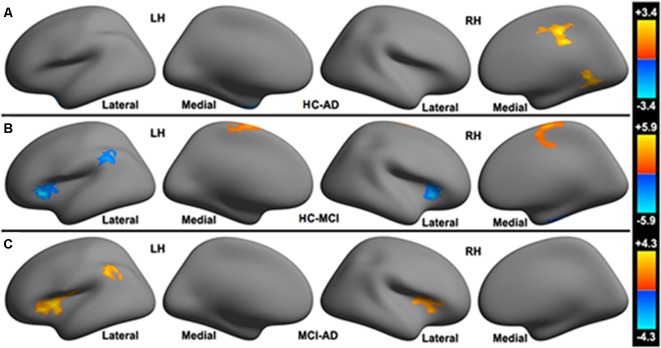
Significant differences in the functional connectivity to the cerebellar limbic network between **(A)** HC, **(B)** MCI and **(C)** AD groups.

HC vs. AD: HC present increased functional connectivity in the right mid-cingulate cortex (MCC), right lingual gyrus to the cerebellar limbic network and decreased functional connectivity in the left temporal pole to cerebellar limbic network in comparison with AD (see Figure 2A).

HC vs. MCI: compared with MCI, HC show increased functional connectivity in the left paracentral lobule, right paracentral lobule extending to the right MCC to the cerebellar limbic network and decreased functional connectivity in the bilateral posterior part of the orbitofrontal cortex (POFC) extending to the anterior insular cortex (AIC), left inferior parietal lobule (L-IPL), and right fusiform gyrus to the cerebellar limbic network (see Figure 2B).

MCI vs. AD: increased functional connectivity between the bilateral POFC extending to the AIC (POFC-AIC), L-IPL and the cerebellar limbic network were found in MCI patients. No decreased functional connectivity was observed in MCI relative to AD (see Figure 2C).

Table 2 illustrated Talairach coordinates of clusters showing significant differences in functional connectivity with the cerebellar limbic network between HC, MCI, and AD groups.

**Table 2:** Talairach coordinates of clusters showing significant differences in functional connectivity with the cerebellar limbic network between HC, MCI, and AD groups.

	Size (mm^2^)	X	Y	Z	BA	Area	T
HC-MCI							
	511	−11	−7	63	6	Left paracentral lobule	1.929
	1,021	13	−16	36	24	Right mid-cingulate cortex/paracentral lobule	3.376
	639	−55	−47	30	40	Left inferior parietal lobule	−1.915
	559	39	22	8		Right posterior part of orbital-fronto cortex and anterior part of insular cortex	−2.554
	499	39	−11	−23	20	Right fusiform gyrus	−2.038
	679	−28	25	5		Left posterior part of orbital-fronto cortex and anterior part of insular cortex	−2.988
HC-AD							
	624	13	−16	36	24	Right mid-cingulate cortex	2.171
	845	13	−64	1		Right lingual gyrus	1.851
	539	−31	−6	−28	20	Left temporal pole	−2.022
MCI-AD							
	1,314	−47	−12	17		Left posterior part of orbital-fronto cortex and anterior part of insular cortex	5.043
	703	−49	−45	43	40	Left inferior parietal lobule	2.192
	734	32	14	10		Right posterior part of orbital-fronto cortex and anterior part of insular cortex	2.865

### Relationships Between Functional Connectivity and MMSE Score

A Pearson correlation was calculated between MMSE score and significant clusters. Among these clusters, the right MCC (HC vs. AD), right lingual gyrus (HC vs. AD), bilateral posterior part of orbital-frontal cortex extending to the AIC (MCI vs. AD), and left inferior parietal lobule (MCI vs. AD) showed positive correlations with the MMSE score (*p* < 0.05). Negative correlation was observed between the left temporal pole (HC vs. AD) and MMSE score (*p* < 0.01; see Table 3). Clusters showing positive or negative correlation with the MMSE score are those with a significant difference in HC vs. AD, and MCI vs. AD. No significant correlations were observed between MMSE score and clusters showing significant differences between HC and MCI patients.

**Table 3:** Significant correlations between the MMSE score and functional connectivity of brain regions.

	R-MCC	R-LG	L-POFC-AIC	L-IPL	R-POFC-AIC	L-TP
Correlation coefficient	0.366	0.366	0.341	0.325	0.313	−0.439
*P*-value	0.017	0.017	0.027	0.036	0.043	0.004

*Note: R-MCC, right mid-cingulate cortex; R-LG, right lingual gyrus; L-POFC-AIC, left posterior part of orbital-fronto cortex and anterior part of insular cortex; L-IPL, left inferior parietal lobule; R-POFC-AIC, right posterior part of orbital-fronto cortex and anterior part of insular cortex; L-TP, left temporal pole*.

## Discussion

The current study evaluated changes in the cerebral limbic network in MCI and AD from the perspective of cerebro-cerebellar functional connectivity because regions of the cerebellum are functionally coupled to specific cerebral networks (Buckner et al., 2011). In other words, we aimed to study the cerebral areas showing significant functional connectivity to the cerebellar limbic network and discriminate differences between HC, MCI, and AD groups. The pattern of the cerebral areas showing functional connectivity to the cerebellar limbic network, which was nearly symmetrically distributed in bilateral hemispheres, was similar in the three groups. The significant differences between the three group indicated that: (1) a sub-scale network, including the bilateral POFC-AIC and left IPL, showed greater functional connectivity in MCI relative to HC and lower connectivity in AD relative to MCI; (2) decreased functional connectivity in the left paracentral lobule and right paracentral lobule extending to right MCC in MCI relative to HC and decreased functional connectivity in the right MCC in AD relative to HC and (3) decreased functional connectivity in the left temporal pole in HC relative to AD and in the right fusiform gyrus in HC relative to MCI, increased functional connectivity in the right lingual gyrus in HC relative to AD. The dual change of the cerebral limbic network in the current study is consistent with a previous study on MCI due to AD patients (Canuet et al., 2015). They found patients with abnormal CSF p-tau and Aβ42 levels showed both decreased and increased functional connectivity affecting limbic structures, with resting-state magnetoencephalography.

The limbic network was proposed by Papez (1995) to be a system involved in emotion and memory. It is well known that AD is characterized by impairment of episodic memory. However, since NPS was verified to occur in AD and MCI (Bruen et al., 2008; Trzepacz et al., 2013), and several previous studies have reported GM atrophy in the limbic network in AD and MCI (Bruen et al., 2008; Trzepacz et al., 2013; Hu et al., 2015), it is reasonable to consider that the limbic network is compromised in AD spectrum disorders.

Although the cerebellum has long been considered to be mainly involved in motor control, evidence has been accumulating that the cerebellum also contributes to higher cognitive function (Buckner, 2013; Bernard and Seidler, 2014; Sokolov et al., 2017; Schmahmann, 2019). Patients with left-sided lesions showed deficits in visuo-spatial processing while patients with right-sided lesions had verbal memory defects, indicating dysfunction of the contralateral cerebral hemispheres (Hokkanen et al., 2006). Other studies related to ADHD (Tomasi and Volkow, 2012), depression (Alalade et al., 2011), and aging (Bernard et al., 2013) without visible parenchymal lesions also revealed that cerebellar function might change accordingly. Moreover, a recent multi-study analysis showed that cerebellar activity during domain-specific mentalizing functionality is strongly connected with a corresponding mentalizing network in the cerebrum (Van Overwalle and Mariën, 2016). However, in the AD spectrum, the cerebellum was considered to be free from Alzheimer pathology until the severe phase of the disease, and always a normal reference in the evaluation of cerebral metabolism (Ni et al., 2013). Bai et al. (2011) reported that longitudinal functional connectivity between the hippocampus subregion and cerebellum may be a valuable biomarker to classify aMCI converters from aMCI non-converters. Similarly, in a recent report (Delli Pizzi et al., 2019), hippocampal/entorhinal functional connectivity were evaluated in individuals with MCI those who did not convert to AD or show the presence of AD pathological load compared to those who converted to AD or showed presence of AD pathological load, increased functional connectivity to the selected regions, especially the cerebellum, were considered to be the strategy in maintaining the cognitive reserve. According to the MMSE and CDR score, AD patients in our study were classified as mild to moderate. It is feasible to illuminate the role of the cerebellum in the evaluation of the limbic network in the AD spectrum by examining coupled cerebro-cerebellar functional connectivity.

### POFC-AIC and IPL

In the current study, the bilateral POFC-AIC, together with the left IPL, formed a sub-scale network that showed a dual pattern in MCI and AD patients. Increased functional connectivity between the sub-scale network and cerebellar limbic network was observed in MCI relative to HC and decreased functional connectivity was observed in AD relative to MCI. The result provided support for a nonlinear trajectory of this sub-scale network activity in the evolution from MCI to AD, similar to the pattern of memory-related neural activity reported by Celone et al. (2006). The OFC and other regions of the prefrontal cortex have been considered important in personality and social behavior. The lateral part of the OFC, which is also a component of limbic cortices, is mainly involved in behavioral inhibition, response inhibition, selective responses, and emotional cognitive adjustment. The OFC is not an area of onset for AD pathology in the brain; however, a large number of neurofibrillary tangles (NFTs) can be observed in the OFC in AD. The density of NFTs in the OFC was only secondary to that of the medial temporal lobe (MTL) even in healthy elderly with normal aging and MCI (Guillozet et al., 2003). These tangles may lead to the changes in OFC activity seen in AD. The anterior insula is an important hub in the emotional limbic system, with the ventral part of anterior insula, just ventral to the primary taste cortex, a part of the limbic system. The ventral anterior insula, together with the anterior cingulate cortex (ACC) and MCC, form the visceromotor limbic cortices. Most researches on the insula have concentrated on its influence on affective disorders. In individuals with depression, task-induced fMRI study showed hypoactivation of the right insula in response to negative affective pictures (Lee et al., 2007), and resting-state fMRI showed decreased Regional Homogeneity (ReHo) in the right insula, which was positively correlated with anxiety severity (Yao et al., 2009). The ventral anterior insular region has strong projections to the OFC and receives inputs from the OFC and ACC (Price, 2007), involving in decoding and representing reward and punishment signals that produce autonomic/visceral responses.

The IPL is not a component of the limbic system and was considered to exhibit strong evidence of a direct role in episodic memory retrieval. Vilberg and Rugg (2008) speculated that the IPL is involved in the maintenance or representation of retrieved information in something like the episodic buffer. In a study of focal lateral parietal damage, Davidson et al. (2008) showed evidence of disrupted recollection in an anterograde memory task. Since some links from the emotional system to the memory system are present, we would like to speculate that the IPL might play a role in limbic activity from the connected network.

The bilateral POFC-AIC and left IPL showed a more positive correlation with the MMSE score in MCI than in AD (*p* < 0.05), whereas no significant difference in correlation was observed between HC and MCI. This indicates that this sub-scale network activity was correlated with Alzheimer pathology, and may be a key biomarker in limbic activity. Moreover, the difference between HC and MCI was much less than that between MCI and AD. Compensatory activity is thought to occur during the progression of AD, especially in individuals at risk of AD (Bookheimer et al., 2000). That is to say, normal-appearing brain areas may be recruited for cognitive activity compensation in the progression of AD (Celone et al., 2006). In Yeo’s Atlas (Yeo et al., 2011), the locations of bilateral POFC-AIC and left IPL are consistent with components of the ventral attention network, so we may speculate that limbic network activity compensation can be verified in MCI through a sub-scale ventral attention network, in addition to decompensation in AD. This coincides with the progress of Alzheimer pathology.

### Paracentral Lobule and MCC

The paracentral lobule and MCC showed a consistent change in MCI and AD relative to HC. The left paracentral lobule and right paracentral lobule extending to right MCC showed compromised functional connectivity to the cerebellar limbic network in MCI, and right MCC in AD. Structural connectivity also showed shorter fibers in the cingulum connecting the MCC with adjacent areas, such as the paracentral lobule, and lingual and fusiform gyri. Generally, the sensorimotor function is spared until severe disease; therefore, the paracentral lobule was not considered to be involved in AD patients in the current study. However, recent reports showed some functional changes in this area, such as increased ReHo index in MCI (Wang et al., 2015), and a significant increase in the nodal centrality in APOE ɛ4 carriers relative to APOE ɛ4 non-carriers based on a graph theory brain network analysis (Yao et al., 2015). Unlike the results of previous reports, the paracentral lobule showed decreased functional connectivity in MCI in this study. The MCC is one part of the limbic cortex, belonging to the hippocampal-centric division, and is also part of the posterior part of the medial DMN. The Pearson correlation indicated that the right MCC showed a more positive correlation with the MMSE score in HC than in AD (*p* < 0.05). No difference in correlation was observed between the right MCC and MMSE scores between HC and MCI, which is analogous to the pattern observed in the POFC-AIC and IPL.

### Fusiform and Lingual Gyri, and Temporal Lobe

The fusiform gyrus is not a component of the cerebral limbic cortices, unlike the lingual gyrus. However, they both participate in mediating the perception of face identity (Hoffman and Haxby, 2000). The fusiform gyrus was also activated in a memory task. With associative encoding of novel picture-word pairs task, MCI subjects showed greater fMRI responses in the fusiform regions, which was believed to be a compensatory change due to the incipient atrophy in the anterior MTL (Hämäläinen et al., 2007). In the present study, the right fusiform showed increased functional connectivity in MCI relative to HC, which was consistent with previous reports. Both the lingual gyrus and temporal pole are parts of the olfactocentric division of the limbic network (Catani et al., 2013). In the comparison between AD and HC, the right lingual gyrus showed decreased while the temporal pole showed increased functional connectivity in AD. However, the correlation analysis showed that a positive correlation with MMSE score was observed in the right lingual gyrus and a negative correlation observed in the right temporal pole. To some degree, the negative correlation between the left temporal pole and MMSE score may indicate that the lower MMSE score in AD patients corresponds to stronger functional activity in this area. Mesulam (1998) has theorized that the temporal poles act as “transmodal epicenters” where information from the multiple sensory modalities is combined to form complex, symbolic, personalized representations. This area was always focused in the study of frontotemporal dementia, especially in the behavioral variant frontotemporal dementia (Hornberger et al., 2011). Zahn et al. (2009) showed that OFC and temporal pole atrophy was associated with disinhibited social behavior. The interaction of the OFC with temporal regions *via* the uncinate fasciculus might be crucial in maintaining normal behavior (Green et al., 2010). A large-scale functional connectivity study showed that the medial part of the left temporal pole is connected to paralimbic structures (Pascual et al., 2015). We should consider that the temporal pole is also a pivotal area in the evaluation of AD and deserves to be studied further.

## Conclusion

The current study investigated the pattern of limbic network activity in the AD spectrum by examining the cerebro-cerebellar functional connectivity as the cerebellum is involved in the modulation of cognitive function. Functional connectivity to the cerebellar limbic network was significantly more compromised in AD than in MCI patients. This coincides with the progress of Alzheimer pathology. However, the dual pattern of the sub-scale ventral attention network, increased in MCI vs. HC and decreased in AD vs. MCI, indicating a compensatory mechanism in the MCI period, may be significant in the illumination of the limbic network in the AD spectrum. And this is consistent with Delli’s report (Delli Pizzi et al., 2019) that increased functional connectivity between the hippocampus and the cerebellar functional associated regions, including the limbic system, was observed in individuals with MCI those who did not convert to AD compared to those who converted to AD. In Skouras’ report (Skouras et al., 2019), cerebellum showed increased functional connectivity to PCC in asymptomatic preclinical AD and to MCC in MCI, these were also considered as functional compensation. Considering that the cerebellum was spared in the early stages of the AD, it is reasonable to believe that the cerebellum is crucial in this process. In addition, the change in left temporal pole activity supports the notion that more attention should be paid to this area in the limbic network in AD.

## Limitation

Although this is a pilot study, we think it was meaningful to investigate the cerebral limbic network activity from the perspective of coupled cerebro-cerebellar functional connectivity. Nonetheless, there were some limitations in this study. The first was the small sample in each group. The second item may be the incomplete neuropsychological study without the use of the Neuropsychiatric Inventory (NPI). Third, we believe it would be more helpful to take the DMN into consideration and analyze the interrelationship between them.

## Data Availability Statement

The raw data supporting the conclusions of this manuscript will be made available by the authors, without undue reservation, to any qualified researcher.

## Ethics Statement

The study was approved by the local ethics committee of Xuanwu Hospital. Written informed consent was obtained from all participants in accordance with the Declaration of Helsinki prior to the study.

## Author Contributions

ZQ, H-JL and JL contributed to the conception and design of the study. ZQ organized the database. H-JL performed the statistical analysis. ZQ, YA and MZ wrote the first draft of the manuscript. All authors contributed to manuscript revision, read and approved the submitted version.

## Conflict of Interest

The authors declare that the research was conducted in the absence of any commercial or financial relationships that could be construed as a potential conflict of interest.

## References

[B1] Alalade E. Denny K. Potter G. Steffens D. Wang L. (2011). Altered cerebellar-cerebral functional connectivity in geriatric depression. PLoS One 6:e20035. 10.1371/journal.pone.0020035 21637831 PMC3102667

[B2] Bai F. Xie C. Watson D. R. Shi Y. Yuan Y. Wang Y.. (2011). Aberrant hippocampal subregion networks associated with the classifications of aMCI subjects: a longitudinal resting-state study. PLoS One 6:e29288. 10.1371/journal.pone.0029288 22216234 PMC3246487

[B3] Bennett M. R. (2011). The prefrontal-limbic network in depression: Modulation by hypothalamus, basal ganglia and midbrain. Prog. Neurobiol. 93, 468–487. 10.1016/j.pneurobio.2011.01.006 21349315

[B4] Bernard J. A. Peltier S. J. Wiggins J. L. Jaeggi S. M. Buschkuehl M. Fling B. W.. (2013). Disrupted cortico-cerebellar connectivity in older adults. Neuroimage 83, 103–119. 10.1016/j.neuroimage.2013.06.042 23792980 PMC3815977

[B5] Bernard J. A. Seidler R. D. (2014). Moving forward: age effects on the cerebellum underlie cognitive and motor declines. Neurosci. Biobehav. Rev. 42, 193–207. 10.1016/j.neubiorev.2014.02.011 24594194 PMC4024443

[B6] Bookheimer S. Y. Strojwas M. H. Cohen M. S. Saunders A. M. Pericak-Vance M. A. Mazziotta J. C.. (2000). Patterns of brain activation in people at risk for Alzheimer’s disease. N. Engl. J. Med. 343, 450–456. 10.1056/NEJM200008173430701 10944562 PMC2831477

[B7] Braak H. Braak E. (1991). Neuropathological stageing of Alzheimer-related changes. Acta Neuropathol. 82, 239–259. 10.1007/bf00308809 1759558

[B8] Bruen P. D. McGeown W. J. Shanks M. F. Venneria A. (2008). Neuroanatomical correlates of neuropsychiatric symptoms in Alzheimer’s disease. Brain 131, 2455–2463. 10.1093/brain/awn151 18669506

[B9] Buckner R. L. (2013). The cerebellum and cognitive function: 25 years of insight from anatomy and neuroimaging. Neuron 80, 807–815. 10.1016/j.neuron.2013.10.044 24183029

[B10] Buckner R. L. Krienen F. M. Castellanos A. Diaz J. C. Yeo B. T. (2011). The organization of the human cerebellum estimated by intrinsic functional connectivity. J. Neurophysiol. 106, 2322–2345. 10.1152/jn.00339.2011 21795627 PMC3214121

[B11] Canuet L. Pusil S. López M. E. Bajo R. Pineda-Pardo J. Á. Cuesta P.. (2015). Network disruption and cerebrospinal fluid amyloid-beta and phospho-tau levels in mild cognitive impairment. J. Neurosci. 35, 10325–10330. 10.1523/jneurosci.0704-15.2015 26180207 PMC6605340

[B12] Catani M. Dell’acqua F. Thiebaut de Schotten M. (2013). A revised limbic system model for memory, emotion and behaviour. Neurosci. Biobehav. Rev. 37, 1724–1737. 10.1016/j.neubiorev.2013.07.001 23850593

[B13] Celone K. A. Calhoun V. D. Dickerson B. C. Atri A. Chua E. F. Miller S. L.. (2006). Alterations in memory networks in mild cognitive impairment and Alzheimer’s disease: an independent component analysis. J. Neurosci. 26, 10222–10231. 10.1523/JNEUROSCI.2250-06.2006 17021177 PMC6674636

[B14] Craig D. Mirakhur A. Hart D. J. McIlroy S. P. Passmore A. P. (2005). A cross-sectional study of neuropsychiatric symptoms in 435 patients with Alzheimer’s disease. Am. J. Geriatr. Psychiatry 13, 460–468. 10.1176/appi.ajgp.13.6.460 15956265

[B15] Davidson P. S. Anaki D. Ciaramelli E. Cohn M. Kim A. S. Murphy K. J.. (2008). Does lateral parietal cortex support episodic memory? Evidence from focal lesion patients. Neuropsychologia 46, 1743–1755. 10.1016/j.neuropsychologia.2008.01.011 18313699 PMC2806230

[B16] Delli Pizzi S. Punzi M. Sensi S. L. Alzheimer’s Disease Neuroimaging Initiative. (2019). Functional signature of conversion of patients with mild cognitive impairment. Neurobiol. Aging 74, 21–37. 10.1016/j.neurobiolaging.2018.10.004 30408719

[B17] Green S. Ralph M. A. Moll J. Stamatakis E. A. Grafman J. Zahn R. (2010). Selective functional integration between anterior temporal and distinct fronto-meso limbic regions during guilt and indignation. Neuroimage 52, 1720–1726. 10.1016/j.neuroimage.2010.05.038 20493953 PMC2941398

[B18] Greicius M. D. Srivastava G. Reiss A. L. Menon V. (2004). Default-mode network activity distinguishes Alzheimer’s disease from healthy aging: evidence from functional MRI. Proc. Natl. Acad. Sci. U S A 101, 4637–4642. 10.1073/pnas.0308627101 15070770 PMC384799

[B19] Guillozet A. L. Weintraub S. Mash D. C. Mesulam M. M. (2003). Neurofibrillary tangles, amyloid, and memory in aging and mild cognitive impairment. Arch. Neurol. 60, 729–736. 10.1001/archneur.60.5.729 12756137

[B20] Habas C. Kamdar N. Nguyen D. Prater K. Beckmann C. F. Menon V.. (2009). Distinct cerebellar contributions to intrinsic connectivity networks. J. Neurosci. 29, 8586–8594. 10.1523/JNEUROSCI.1868-09.2009 19571149 PMC2742620

[B21] Hämäläinen A. Pihlajamäki M. Tanila H. Hänninen T. Niskanen E. Tervo S.. (2007). Increased fMRI responses during encoding in mild cognitive impairment. Neurobiol. Aging 28, 1889–1903. 10.1016/j.neurobiolaging.2006.08.008 16997428

[B22] Hoffman E. A. Haxby J. V. (2000). Distinct representations of eye gaze and identity in the distributed human neural system for face perception. Nat. Neurosci. 3, 80–84. 10.1038/71152 10607399

[B23] Hokkanen L. S. K. Kauranen V. Roine R. O. Salonen O. Kotila M. (2006). Subtle cognitive deficits after cerebellar infarcts. Eur. J. Neurol. 13, 161–170. 10.1111/j.1468-1331.2006.01157.x 16490047

[B24] Hornberger M. Geng J. Hodges J. R. (2011). Convergent grey and white matter evidence of orbitofrontal cortex changes related to disinhibition in behavioural variant frontotemporal dementia. Brain 134, 2502–2512. 10.1093/brain/awr173 21785117

[B25] Hu X. Meiberth D. Newport B. Jessen F. (2015). Anatomical correlates of the neuropsychiatric symptoms in Alzheimer’s disease. Curr. Alzheimer Res. 12, 266–277. 10.2174/1567205012666150302154914 25731626

[B26] Khan U. A. Liu L. Provenzano F. A. Berman D. E. Profaci C. P. Sloan R.. (2014). Molecular drivers and cortical spread of lateral entorhinal cortex dysfunction in preclinical Alzheimer’s disease. Nat. Neurosci. 17, 304–311. 10.1038/nn.3606 24362760 PMC4044925

[B27] Lee B. T. Cho S. W. Khang H. S. Lee B. C. Choi I. G. Lyoo I. K.. (2007). The neural substrates of affective processing toward positive and negative affective pictures in patients with major depressive disorder. Prog. Neuropsychopharmacol. Biol. Psychiatry 31, 1487–1492. 10.1016/j.pnpbp.2007.06.030 17688985

[B28] Leow A. Ajilore O. Zhan L. Arienzo D. GadElkarim J. Zhang A.. (2013). Impaired inter-hemispheric integration in bipolar disorder revealed with brain network analyses. Biol. Psychiatry 73, 183–193. 10.1016/j.biopsych.2012.09.014 23122540 PMC4113030

[B29] Li H. J. Hou X. H. Liu H. H. Yue C. L. He Y. Zuo X. N. (2015). Toward systems neuroscience in mild cognitive impairment and Alzheimer’s disease: a meta-analysis of 75 fMRI studies. Hum. Brain Mapp. 36, 1217–1232. 10.1002/hbm.22689 25411150 PMC6869191

[B30] Lu J. Liu H. Zhang M. Wang D. Cao Y. Ma Q.. (2011). Focal pontine lesions provide evidence that intrinsic functional connectivity reflects polysynaptic anatomical pathways. J. Neurosci. 31, 15065–15071. 10.1523/jneurosci.2364-11.2011 22016540 PMC3397237

[B31] Mesulam M. M. (1998). From sensation to cognition. Brain 121, 1013–1052. 10.1093/brain/121.6.1013 9648540

[B32] Nestor P. J. Fryer T. D. Smielewski P. Hodges J. R. (2003). Limbic hypometabolism in Alzheimer’s disease and mild cognitive impairment. Ann. Neurol. 54, 343–351. 10.1002/ana.10669 12953266

[B33] Ni R. Gillberg P. G. Bergfors A. Marutle A. Nordberg A. (2013). Amyloid tracers detect multiple binding sites in Alzheimer’s disease brain tissue. Brain 136, 2217–2227. 10.1093/brain/awt142 23757761

[B34] O’Reilly J. X. Beckmann C. F. Tomassini V. Ramnani N. Johansen-Berg H. (2010). Distinct and overlapping functional zones in the cerebellum defined by resting state functional connectivity. Cereb. Cortex 20, 953–965. 10.1093/cercor/bhp157 19684249 PMC2837094

[B35] Papez J. W. (1995). A proposed mechanism of emotion. J. Neuropsychiatry Clin. Neurosci. 7, 103–112. 10.1176/jnp.7.1.103 7711480

[B36] Pascual B. Masdeu J. C. Hollenbeck M. Makris N. Insausti R. Ding S. L.. (2015). Large-scale brain networks of the human left temporal pole: a functional connectivity MRI study. Cereb. Cortex 25, 680–702. 10.1093/cercor/bht260 24068551 PMC4318532

[B37] Price J. L. (2007). Definition of the orbital cortex in relation to specific connections with limbic and visceral structures and other cortical regions. Ann. N Y Acad. Sci. 1121, 54–71. 10.1196/annals.1401.008 17698999

[B38] Pruessner J. C. Dedovic K. Khalili-Mahani N. Engert V. Pruessner M. Buss C.. (2008). Deactivation of the limbic system during acute psychosocial stress: evidence from positron emission tomography and functional magnetic resonance imaging studies. Biol. Psychiatry 63, 234–240. 10.1016/j.biopsych.2007.04.041 17686466

[B39] Rolls E. T. (2015). Limbic systems for emotion and for memory, but no single limbic system. Cortex 62, 119–157. 10.1016/j.cortex.2013.12.005 24439664

[B40] Schmahmann J. D. (2019). The cerebellum and cognition. Neurosci. Lett. 688, 62–75. 10.1016/j.neulet.2018.07.005 29997061

[B41] Skouras S. Falcon C. Tucholka A. Rami L. Sanchez-Valle R. Lladó A.. (2019). Mechanisms of functional compensation, delineated by eigenvector centrality mapping, across the pathophysiological continuum of Alzheimer’s disease. Neuroimage Clin. 22:101777. 10.1016/j.nicl.2019.101777 30913531 PMC6434094

[B42] Sokolov A. A. Miall R. C. Ivry R. B. (2017). The cerebellum: adaptive prediction for movement and cognition. Trends Cogn. Sci. 21, 313–332. 10.1016/j.tics.2017.02.005 28385461 PMC5477675

[B43] Spires-Jones T. L. Hyman B. T. (2014). The intersection of amyloid beta and tau at synapses in Alzheimer’s disease. Neuron 82, 756–771. 10.1016/j.neuron.2014.05.004 24853936 PMC4135182

[B45] Tomasi D. Volkow N. D. (2012). Abnormal functional connectivity in children with attention-deficit/hyperactivity disorder. Biol. Psychiatry 71, 443–450. 10.1016/j.biopsych.2011.11.003 22153589 PMC3479644

[B46] Trzepacz P. T. Yu P. Bhamidipati P. K. Willis B. Forrester T. Tabas L.. (2013). Frontolimbic atrophy is associated with agitation and aggression in mild cognitive impairment and Alzheimer’s disease. Alzheimers Dement. 9, S95.e1–S104.e1. 10.1016/j.jalz.2012.10.005 23253778 PMC3955297

[B47] Van Overwalle F. Mariën P. (2016). Functional connectivity between the cerebrum and cerebellum in social cognition: a multi-study analysis. Neuroimage 124, 248–255. 10.1016/j.neuroimage.2015.09.001 26348560

[B48] Vilberg K. L. Rugg M. D. (2008). Memory retrieval and the parietal cortex: review of evidence from a dual-process perspective. Neuropsychologia 46, 1787–1799. 10.1016/j.neuropsychologia.2008.01.004 18343462 PMC2488316

[B49] Wang Y. Zhao X. Xu S. Yu L. Wang L. Song M.. (2015). Using regional homogeneity to reveal altered spontaneous activity in patients with mild cognitive impairment. Biomed Res. Int. 2015:807093. 10.1155/2015/807093 25738156 PMC4337114

[B50] Winblad B. Palmer K. Kivipelto M. Jelic V. Fratiglioni L. Wahlund L. O.. (2004). Mild cognitive impairment-beyond controversies, towards a consensus: report of the international working group on mild cognitive impairment. J. Intern. Med. 256, 240–246. 10.1111/j.1365-2796.2004.01380.x 15324367

[B51] Xing X. X. Zhou Y. L. Adelstein J. S. Zuo X. N. (2011). PDE-based spatial smoothing: a practical demonstration of impacts on MRI brain extraction, tissue segmentation and registration. Magn. Reson. Imaging 29, 731–738. 10.1016/j.mri.2011.02.007 21531104

[B44] Xu T. Yang Z. Jiang L. Xing X.-X. Zuo X.-N. (2015). A Connectome Computation System for discovery science of brain. Sci. Bull. 60, 86–95. 10.1007/s11434-014-0698-3

[B52] Yao Z. Hu B. Zheng J. Zheng W. Chen X. Gao X.. (2015). A FDG-PET study of metabolic networks in Apolipoprotein E ε4 allele carriers. PLoS One 10:e0132300. 10.1371/journal.pone.0132300 26161964 PMC4498596

[B53] Yao Z. Wang L. Lu Q. Liu H. Teng G. (2009). Regional homogeneity in depression and its relationship with separate depressive symptom clusters: a resting-state fMRI study. J. Affect. Disord. 115, 430–438. 10.1016/j.jad.2008.10.013 19007997

[B54] Yeo B. T. Krienen F. M. Sepulcre J. Sabuncu M. R. Lashkari D. Hollinshead M.. (2011). The organization of the human cerebral cortex estimated by intrinsic functional connectivity. J. Neurophysiol. 106, 1125–1165. 10.1152/jn.00338.2011 21653723 PMC3174820

[B55] Zahn R. Moll J. Iyengar V. Huey E. D. Tierney M. Krueger F.. (2009). Social conceptual impairments in frontotemporal lobar degeneration with right anterior temporal hypometabolism. Brain 132, 604–616. 10.1093/brain/awn343 19153155 PMC2724922

[B56] Zhang Y. W. Zhao Z. L. Qi Z. Hu Y. Wang Y. S. Sheng C.. (2017). Local-to-remote cortical connectivity in amnestic mild cognitive impairment. Neurobiol. Aging 56, 138–149. 10.1016/j.neurobiolaging.2017.04.016 28528774

